# Perceptual load modulates the delta oscillation and the contribution of delta oscillation to reward positivity during feedback valence encoding

**DOI:** 10.3389/fpsyg.2025.1658756

**Published:** 2025-08-15

**Authors:** Congcong Qi, Jinming Zhu, Lele Chen, Lingyao Tong, Shiwei Jia

**Affiliations:** ^1^Faculty of Psychology, Shandong Normal University, Jinan, China; ^2^Shandong Provincial Key Laboratory of Brain Science and Mental Health, Jinan, China; ^3^Mental Health Education Center, Shandong First Medical University, Jinan, China; ^4^Department of Clinical, Neuro and Developmental Psychology, Amsterdam Public Health Research Institute, Vrije Universiteit Amsterdam, Amsterdam, Netherlands

**Keywords:** feedback processing, perceptual load, RewP, P300, delta oscillation, theta oscillation, reward processing

## Abstract

RewP (reward positivity) and P300 are feedback-related ERP components, while delta and theta are oscillatory responses evoked during feedback processing. While the perceptual difficulty of feedback modulates ERP components, its impact on feedback-related oscillatory activities remains unexplored. In this study, the perceptual load of feedback stimuli was manipulated to investigate its influence on the valence effect of both ERP and oscillatory components. Further, the contribution of the oscillations’ valence effect (e.g., the difference wave between positive and negative feedback) to the valence effect of ERP components was analyzed, alongside the moderation of perceptual load on these contributions. The results indicated that, for both the RewP and P300, the amplitudes evoked by positive feedback were greater than those evoked by negative feedback. For theta oscillation, however, the activity evoked by negative feedback was stronger than that evoked by positive feedback. These valence effects were unaffected by perceptual load. However, for delta oscillation, its valence effect was modulated by perceptual load, only under low-load conditions did positive feedback elicit greater delta activity than negative feedback. The correlation analysis for the difference wave between positive and negative feedback showed that the RewP was significantly correlated with the P300, while delta and theta activities were not significantly correlated. The regression analysis of the difference wave revealed that delta significantly predicted the RewP under low-load conditions, while theta significantly predicted the P300 under high-load conditions. These results suggest that delta and theta oscillations reflect the processing of positive and negative feedback, respectively. Perceptual load modulates only positive feedback processing; the lower the load, the easier the processing. Perceptual load also modulates the contribution of delta oscillation to the RewP, but not to the P300.

## 1 Introduction

Feedback processing allows individuals to determine whether their behavior is appropriate, facilitating the maintenance of appropriate behavior, the adjustment of inappropriate behavior, and better adaptation to the environment (Thorndike, 1911; Holroyd and Coles, 2002; Walsh and Anderson, 2012; Luft, 2014). Feedback processing and learning constitute an ongoing daily process of perception and adaptation. Recent electrophysiological studies have identified key components related to feedback processing, including the ERP components of RewP (reward positivity) and P300 (Yeung and Sanfey, 2004; Leng and Zhou, 2010; Glazer et al., 2018; Zhang et al., 2023; Bauer et al., 2024), as well as oscillations in the delta and theta bands (Bernat et al., 2011, 2015; Foti et al., 2015; Szenczy et al., 2025). The identification of these components has significantly advanced neurophysiological research on feedback processing and learning (Holroyd and Coles, 2002; Frank et al., 2005; Luft, 2014; Holroyd and Umemoto, 2016; Walsh and Anderson, 2012).

The perceptual difficulty of feedback stimuli affects feedback perception, and consequently, it alters the neurophysiological signals associated with feedback processing. Researchers have manipulated various aspects of feedback stimuli, such as their perceptual properties (Liu and Gehring, 2009; Liu et al., 2014; Pfabigan et al., 2015) and the amount of information they convey (Mars et al., 2004; Cockburn and Holroyd, 2018), to examine the influence of perceptual difficulty on feedback-related ERP components, which will be introduced in the following.

### 1.1 The feedback processing related electrophysiological components

The electrophysiological components associated with feedback processing include the ERP components RewP and P300 (Proudfit, 2015; Glazer et al., 2018), as well as oscillations in the delta (1–3 Hz) and theta (4–7 Hz) frequency bands (Bernat et al., 2008, 2011, 2015; Foti et al., 2015; Bowers et al., 2018). The following section will describe each of these components in detail.

RewP typically appears 250–350 ms after feedback onset and is distributed across the middle and frontal scalp regions. Positive feedback (such as monetary gain or correct behavior) induces a more positive component than negative feedback (monetary loss or incorrect behavior) (Walsh and Anderson, 2012; Sambrook and Goslin, 2016; Proudfit, 2015). Source localization and neuroimaging studies suggest that RewP originates from the anterior cingulate cortex (ACC) and/or the striatum (Miltner et al., 1997; Gehring and Willoughby, 2002; Hauser et al., 2014; Becker et al., 2014; Glazer et al., 2018). Initially, this component was thought to reflect negative feedback processing and was referred to as the FRN (Feedback-related negativity, Nieuwenhuis, 2004). Recent studies have revealed that the difference wave between positive and negative feedback is associated with a positive component induced by positive feedback. As a result, the FRN was renamed RewP and is now considered an indicator of reward sensitivity in psychopathology research (Proudfit, 2015; Paul et al., 2025).

P300 is another crucial ERP component elicited by feedback, appearing immediately after RewP and distributed across the middle and posterior scalp regions. Studies have shown that P300 reflects the integration of background information and the updating of working memory. Its amplitude is related to cognitive resource allocation, serving as an indicator of resource input (Polich, 2007; Nieuwenhuis et al., 2005; Paul et al., 2025). Some studies suggest that P300 is sensitive to feedback magnitude but not to feedback valence (Yeung and Sanfey, 2004; Sato et al., 2005). Other studies have found that positive feedback elicits a greater P300 response than negative feedback and that “action” enhances the valence effect of P300 (Zhou et al., 2010). Another study manipulated interpersonal relationship closeness and found that self-feedback and feedback from friends elicited a greater P300 valence effect compared to feedback from strangers (Leng and Zhou, 2010). One study found that feedback interval can affect the P300 valence effect, with a reduction in the valence effect during longer intervals (Wang et al., 2014). Therefore, the valence effect of P300 remains inconsistent across existing studies. Compared to RewP, P300 is more sensitive to complex feedback information.

The introduction of RewP reflects a change in the understanding of its function. Initially, it was believed to be a negative component, reflecting error or loss detection, but it was later recognized as a positive component associated with reward processing. Neither perspective fully accounts for the evaluation of both positive and negative feedback simultaneously. Through time-frequency analysis, researchers have identified two oscillations, delta and theta (Bernat et al., 2005, 2011). Delta oscillation ranges from 1 to 3 Hz and shows increased activity following monetary gain, with peak activity observed at the middle and posterior scalp regions. Theta oscillation ranges from 4 to 7 Hz and increases following monetary loss, with peak activity observed at the middle and frontal scalp regions. Foti et al. (2015) also found that theta and delta oscillations are sensitive to monetary loss and gain, respectively, with theta reflecting ACC activity and delta reflecting basal ganglia activity. Foti et al. (2015) suggest that RewP integrates positive and negative feedback-related oscillations. Therefore, time-frequency analysis can complement ERP findings.

The relationship between ERP components and oscillations has also been examined. Watts et al. (2017) found significant correlations between RewP and P300, as well as between delta and theta. However, further analysis using the difference wave between positive and negative feedback (valence effect) revealed that RewP was significantly correlated with P300, whereas delta and theta were not significantly correlated (Bernat et al., 2011, 2015). This suggests that RewP and P300 share some common activity, while delta and theta are more independent and reflect distinct cognitive processes. Regression analysis revealed that both delta and theta independently contribute to RewP, but in opposite directions. The valence effects of delta and theta contribute to the valence effect of RewP in an additive manner, suggesting that RewP is sensitive to both negative and positive feedback evaluations. Moreover, both delta and theta can independently contribute to P300 (Watts et al., 2017; Bernat et al., 2011, 2015). However, the valence effects of delta and theta offset each other in contributing to the valence effect of P300, resulting in an unstable valence effect of P300 across previous studies.

Moreover, an increasing number of studies have proposed that the RewP is not a standalone ERP component but rather reflects the integration of distinct oscillatory activities. Time-frequency analyses have revealed that delta (1–3 Hz) activity is predominantly associated with positive feedback, whereas theta (4–7 Hz) activity is linked to negative feedback (Bernat et al., 2011; Foti et al., 2015). These findings suggest that the RewP may arise from the superposition or interaction of underlying delta and theta oscillations. Thus, understanding how perceptual load affects these oscillations can clarify whether changes in RewP are driven by modulations in specific frequency bands. This perspective provides a theoretical rationale for linking ERP and time-frequency analyses in the present study.

### 1.2 Perceptual difficulty affects the feedback-related electrophysiological responses

Previous studies have revealed that perceptual difficulty influences the RewP through various means, which can be grouped into three main categories: perceptual features of stimuli (e.g., color, size, feature complexity); the amount of information or cognitive calculation required; and presentation formats such as blocked versus randomized feedback. These studies consistently indicate that higher perceptual load tends to reduce or eliminate the RewP effect.

Several studies have demonstrated that the physical characteristics of feedback stimuli significantly affect feedback processing. For instance, Liu and Gehring (2009) compared single-feature (e.g., color only) versus combined-feature (e.g., color and shape) feedback in a gambling task and found that single-feature feedback elicited a stronger RewP effect. Extending this line of inquiry, Liu et al. (2014) manipulated feature similarity and congruency between feedback and flankers. RewP was more pronounced when feedback stimuli were dissimilar (e.g., S vs. T) and congruent (e.g., EEEEE), suggesting easier perceptual discrimination enhances reward-related neural responses. Pfabigan et al. (2015) further confirmed this by showing that larger-sized feedback stimuli evoked a stronger RewP. Collectively, these findings indicate that perceptually salient and easily discriminable feedback enhances neural sensitivity to feedback valence.

Other research has focused on how the amount and complexity of information embedded in the feedback modulate RewP. Mars et al. (2004) systematically increased the amount of evaluative content in feedback (e.g., correctness, direction, magnitude) and observed a progressive attenuation of the RewP as information load increased. Cockburn and Holroyd (2018) replicated this result, concluding that excessive informational content reduces evaluative efficiency, weakening reward-related ERP components. Similarly, Gehring and Willoughby (2002) and Nieuwenhuis (2004) found that feedback requiring computation (e.g., calculating which number was “better”) failed to elicit a RewP, while color-coded gain/loss feedback did. Krigolson et al. (2012) also showed that when feedback interpretation required mental arithmetic (e.g., summing integers), the RewP effect disappeared. These studies underscore that cognitive load during feedback evaluation dampens reward-related electrophysiological responses.

Finally, some studies have examined how the format in which feedback is presented affects RewP. Gibbons et al. (2016) used a pseudo-learning task with word-pair feedback presented either in fixed blocks or randomly trial-by-trial. Stronger RewP effects were found in the blocked condition, likely due to increased processing fluency. Pfabigan et al. (2014) found similar results using facial expressions and symbols as feedback stimuli: block-fixed presentation elicited stronger RewP than randomized presentation. The authors suggest that randomized formats hinder the automaticity of feedback processing, increasing perceptual difficulty and thus diminishing reward-related neural responses. Together, these studies highlight the influence of stimulus consistency and expectation on feedback evaluation.

### 1.3 The current study: the effect of perceptual load on the feedback-related electrophysiological components

Feedback processing and learning involve multiple cognitive stages, including the perceptual stage as well as the learning and memory stages (Luft, 2014), during which, cognitive control and attention factors are required for behavioral adjustments (Holroyd and Umemoto, 2016). Focusing studies on specific cognitive stages or factors one at a time can help reveal the internal mechanisms of feedback processing and learning progressively. This study focuses on the perceptual stage.

In Section 1.2, we reviewed studies that have manipulated the perceptual stage of feedback processing, which generally found that as perceptual difficulty increases, the RewP effect diminishes or even disappears. However, the effects of perceptual difficulty on oscillatory components remain unexplored. This study manipulates perceptual load to investigate its impact on feedback perception, specifically focusing on both ERP and oscillatory components.

A simple gambling task with a cross-manipulation feedback design was used in this study. High and low perceptual load conditions were achieved through this cross-manipulation of feedback. Specifically, feedback was presented as a horizontal line and a vertical line in a cross manner. The two lines could be distinguished along two dimensions: color and relative length. The color judgment was easy, indicating low perceptual load, while the relative length judgment was more difficult, indicating high perceptual load (Lavie, 2006; Cartwright-Finch and Lavie, 2007). In this study, feedback was conveyed by the line length in the high-load condition and the line color in the low-load condition. Previous studies have shown that this cross-manipulation affects perceptual load specifically, without altering the working memory load in the two conditions (Cartwright-Finch and Lavie, 2007; Brockhoff et al., 2022), making it an ideal method for the current study.

Compared to ERP components like RewP and P300, delta and theta oscillations are more independent and can better distinguish between feedback processes (Bernat et al., 2015; Watts et al., 2017). Analyzing both ERP and oscillatory components together provides complementary insights into a single cognitive stage. Therefore, this study analyzes both ERP and oscillatory components. Moreover, to further clarify the functions of ERP and oscillatory components and interpret the results, correlation analyses between RewP and P300, delta and theta, as well as regression analyses using oscillatory components to predict ERP components, were conducted.

Based on prior findings, we formulated the following directional hypotheses:

RewP: We expected a stronger RewP amplitude in response to positive compared to negative feedback, with the valence effect enhanced under low perceptual load. P300: We predicted larger P300 amplitudes for positive than negative feedback, and overall greater P300 under low load. However, we did not expect a strong modulation of the valence effect by load, as P300 is associated with post-perceptual processes.

Delta oscillation: We expected greater delta power for positive compared to negative feedback, and that this valence effect would be more pronounced under low perceptual load. Theta oscillation: We predicted greater theta power for negative than positive feedback, with no strong expectation for interaction with perceptual load, due to prior inconsistencies.

In correlational analyses, we anticipated that the valence effects of RewP and P300 would be positively correlated, while delta and theta would not be significantly correlated (Watts et al., 2017). In regression analyses, we expected delta and theta to independently predict RewP, and potentially P300 (Bernat et al., 2015). We explored whether perceptual load would moderate these regression relationships, but made no strong a priori predictions on this aspect.

## 2 Methods

### 2.1 Participants

Twenty-five participants (14 females), aged 18 to 25 years (*M* = 21 ± 3.2), were recruited for this study. All participants were right-handed, had normal or corrected-to-normal vision, and had normal color vision. They had no history of mental illness, head trauma, or recent use of psychoactive substances. The sample size was calculated using PANGE (https://jakewestfall.shinyapps.io/pangea). To achieve a large effect size (Cohen’s *d* = 0.80) with a statistical power of 85%, the required sample size was determined to be 17 participants. Thus, 25 participants met the sample size requirement. This study was approved by the Ethics Committee of Shandong Normal University.

### 2.2 Experimental task

**Figure 1:**
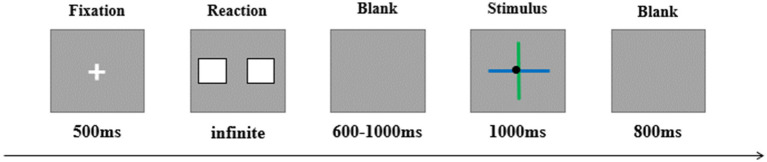
Illustration of one trial. In this trial, the vertical line is short and green. In the low-load condition, participants evaluate the feedback based on the color of the vertical line; in the high-load condition, participants evaluate the feedback based on the length of the vertical line.

This study employed a 2 × 2 design, with feedback valence (positive, negative) and perceptual load (high, low) as two within-participant factors. In a simple gambling task, feedback was presented using a cross-manipulation method to achieve high and low perceptual load conditions. Specifically, the experiment followed the cross-paradigm of Cartwright-Finch and Lavie (2007) to present the feedback. As shown in Figure 1, the feedback consisted of two lines crossing each other, with the lines differing in two dimensions: color and relative length. The color dimension provided a clear contrast, with one line green (RGB: 0, 234, 41) and the other blue (RGB: 0, 191, 255). The length dimension, while less contrasting, could still be discriminated, with one line being relatively long (visual angle: 3.89°) and the other relatively short (visual angle: 3.45°). The point where the two lines crossed was marked by a black dot (Cartwright-Finch and Lavie, 2007). Feedback valence was represented by the vertical line. In the low-load condition, the color of the vertical line indicated gain or loss, with the meanings of blue and green counterbalanced across participants. In the high-load condition, the relative length of the vertical line indicated gain or loss, with the meanings of the line lengths also counterbalanced. Positive and negative feedback were presented randomly.

During the experiment, participants sat in a soundproof, well-lit room. Each trial began with a 500 ms fixation, followed by the presentation of two cards. Participants were instructed to choose one card to gamble on, with the “F” key used to select the left card and the “J” key to select the right card. They were required to make a choice within 3 s. After the choice, a blank screen appeared for a random duration between 600 and 1,000 ms, followed by 1,000 ms of feedback. The inter-trial interval was 800 ms.

The experiment consisted of 4 blocks, each containing 80 trials. At the beginning of the task, participants practiced with at least 20 trials to familiarize themselves with the cross-feedback design. They were informed that there were certain rules linking their responses to the feedback and encouraged to explore these rules to improve their performance. In reality, no such rule existed, and feedback (both positive and negative) was presented randomly. At the end of the experiment, the purpose of the study and the pseudo-random design were explained to the participants, and they were compensated for their participation.

### 2.3 Data recording and analysis

The experiment was conducted using E-Prime 2.0 (Psychology Software Tools, Inc., Sharpsburg, PA) and was presented on a high-performance monitor (Zhang et al., 2018). EEG data were recorded from 64 electrodes mounted in an elastic cap according to the 10–20 system, and amplified using a Brain Products system (Brain Products GmbH, Munich, Germany). The vertical electrooculogram (VEOG) was recorded from an electrode placed 1.5 cm below the right eye’s orbit, and the horizontal electrooculogram (HEOG) was recorded from an electrode 1.5 cm outside the left eye’s orbit. The signals were amplified with a band-pass filter of 0.016–70 Hz and digitized at a sampling rate of 1,000 Hz. All electrodes were referenced to FCz with AFz serving as the ground electrode, and electrode impedances were kept below 5 kΩ.

Offline analysis was performed using EEGLAB (Delorme and Makeig, 2004) in MATLAB R2021a. The sampling rate was reduced to 500 Hz, and EEG data were corrected for horizontal and vertical EOG artifacts using independent component analysis (ICA). The data were then re-referenced to the average of all electrodes, followed by a band-pass filter of 0.5–30 Hz. Artifacts with amplitudes greater than ±100 μV were rejected. EEG signals were segmented from 200 ms before to 1,000 ms after feedback onset, with the 200 ms pre-feedback interval used as the baseline.

The RewP and P300 amplitudes were measured based on the grand average waveforms, following the procedures outlined in previous literature (Glazer et al., 2018). The mean amplitude of the RewP was calculated from 270 ms to 330 ms at the Fz, FCz, and Cz electrodes, and the average across these three electrodes was used to represent the RewP amplitude. The mean amplitude of the P300 was measured from 330 ms to 410 ms at the Cz and CPz electrodes, with the average of these two electrodes serving as the P300 amplitude.

For time-frequency analysis, we used continuous wavelet transform (CWT) in Letswave, following the approach outlined by Webb et al. (2017). To minimize edge effects, EEG data were segmented from 1,000 ms before to 1,500 ms after feedback onset, and the complex Morlet wavelet transform was applied to this segment. The center frequency (*ω*) and limit (*σ*) parameters were set to 1.5 and 1, respectively. The frequency range from 0.5–30 Hz was decomposed into 100 linearly spaced bins, providing sufficient resolution to isolate activity in delta (<3 Hz) and theta (4–7 Hz) bands while ensuring computational efficiency. To quantify power modulation, we computed event-related spectral perturbation (ERSP), defined as the log ratio between signal power at each time-frequency point and the mean baseline power from −500 to −300 ms before feedback onset. This normalization method eliminates inter-subject baseline variability and reduces the influence of edge artifacts. The data were then averaged across different conditions, and the time window of 200 ms before and 800 ms after feedback onset was defined as the analysis window.

Next, ERSP was calculated for each frequency band using the formula:


$$ER_{t,f}\%=\frac{A_{t,f}-R_{f}}{R_{f}}$$

Where $A_{\left\{t,f\right\}}$ represents the signal energy at a specific time *t* and frequency band *f*, and *R_f_* represents the average baseline energy for the frequency band *f* (Pfurtscheller and Da Silva, 1999). To minimize the impact of edge artifacts during wavelet transformation (Cohen and Cavanagh, 2011), the baseline for ERSP calculation was taken from 300 ms to 500 ms before feedback onset. Delta activity was measured from 250 ms to 450 ms after feedback onset at the Cz electrode, while theta activity was measured from 250 ms to 450 ms after feedback onset at the FCz electrode.

For the analysis of ERP components, a 2 (perceptual load: low, high) × 2 (valence: positive, negative) two-factor repeated measures ANOVA was conducted on both RewP and P300. Additionally, the RewP for the negative condition was subtracted from that for the positive condition to obtain the RewP difference wave. A t-test was then conducted to compare the difference waves between high and low perceptual load conditions. The analysis of the time-frequency data followed the same procedure as the ERP analysis, with the same two-factor repeated measures ANOVA conducted on both delta and theta oscillations.

## 3 Results

### 3.1 ERP results

**Figure 2:**
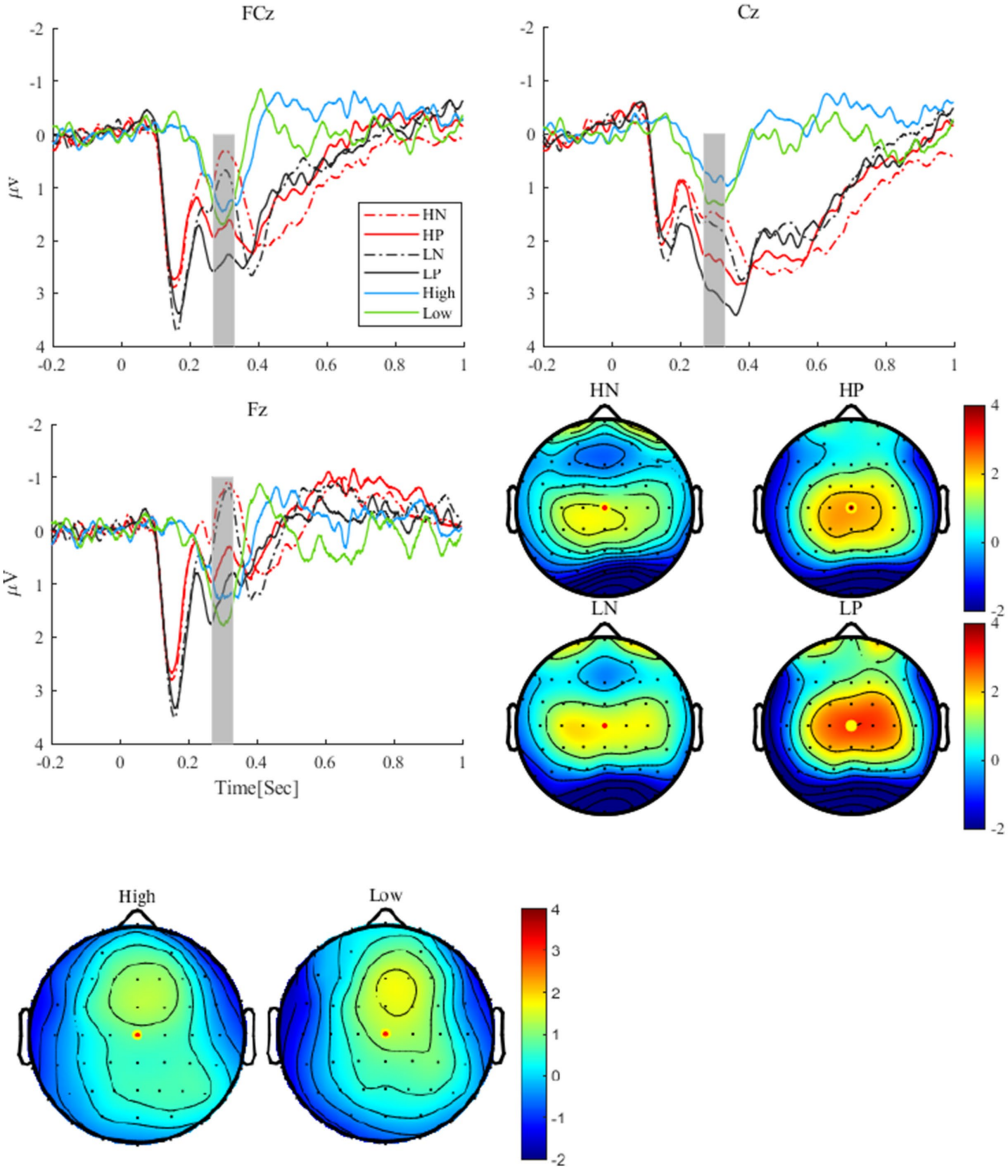
The grand average waveforms and difference waves of RewP at FCz, Cz, and Fz electrodes, and RewP topographies under different conditions, high and low were the topographies **(A)** the difference waves **(B)**. HN, high perceptual load negative feedback; HP, high perceptual load positive feedback; LN, low perceptual load negative feedback; LP, low perceptual load positive feedback.

As shown in Figure 2, for the RewP, the main effect of valence was significant, *F*(1, 24) = 25.27, *p* < 0.001, *η2 p* = 0.51, with the RewP in the positive feedback condition (2.63 ± 0.28 *μV*) being more positive than that in the negative feedback condition (1.51 ± 0.27 *μV*). The main effect of perceptual load was also significant, *F*(1, 24) = 5.45, *p* = 0.028, *η2 p* = 0.19. The RewP in low perceptual load condition (2.34 ± 0.29 *μV*) was more positive than that in high perceptual load condition (1.79 ± 0.26 *μV*). The interaction between perceptual load and valence was not significant (*p* = 0.775). For the RewP difference wave, the *t*-test revealed no significant difference between the high and low perceptual load conditions, *t* (24) = 1.52, *p* = 0.142.

**Figure 3:**
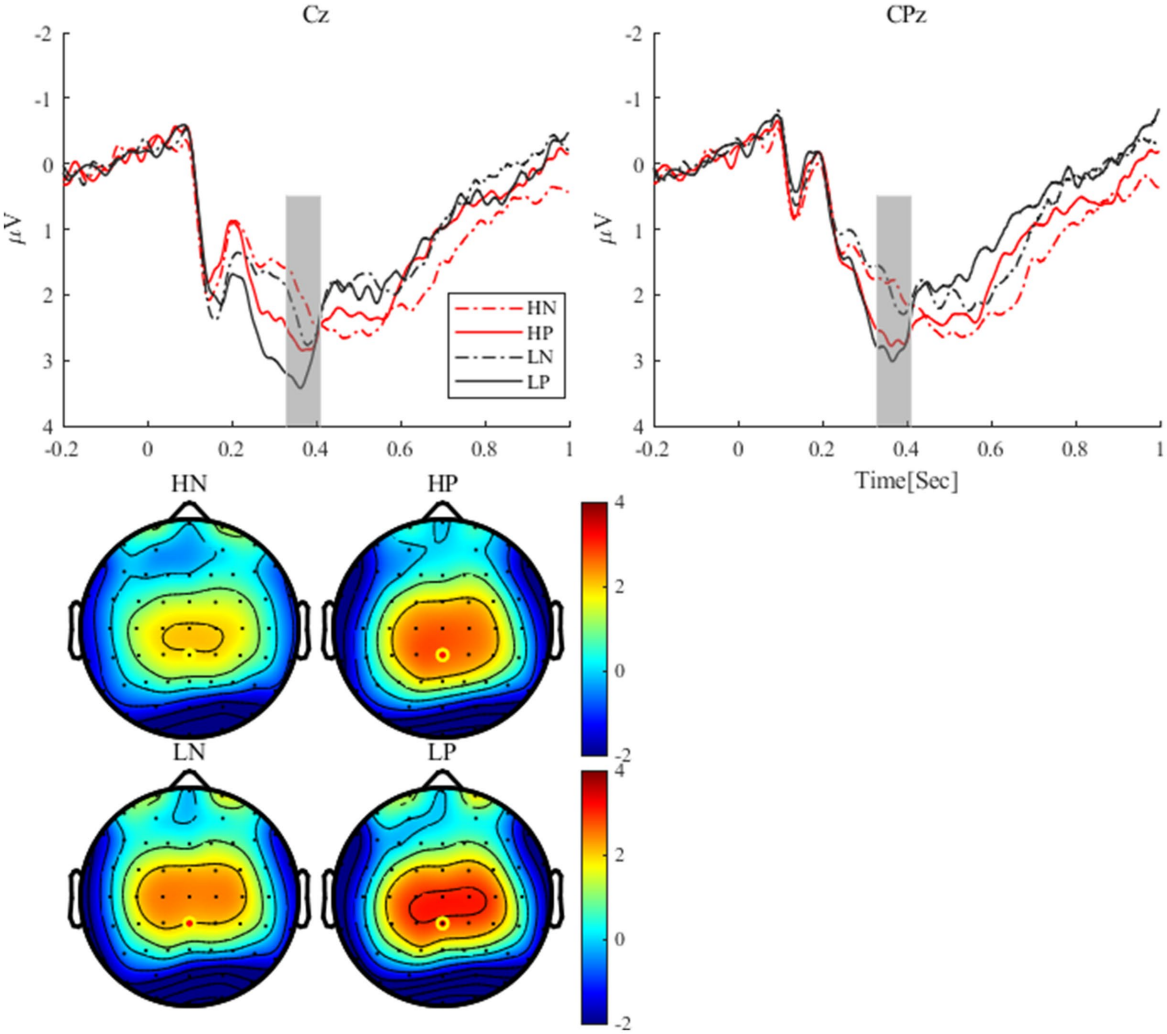
The P300 waveforms at Cz and CPz electrodes and P300 topographies under different conditions.

As shown in Figure 3, for the P300, the main effect of valence was significant, *F*(1, 24) = 11.47, *p* = 0.002, *η2 p* = 0.32, the P300 after positive feedback (3.54 ± 0.37 *μV*) was more positive than that after negative feedback (2.90 ± 0.27 *μV*). The main effect of perceptual load was significant, *F*(1, 24) = 8.88, *p* = 0.007, *η2 p* = 0.27. The P300 under low perceptual load condition (3.46 ± 0.33 *μV*) was more positive than that under high perceptual load condition (2.90 ± 0.27 *μV*). The interaction between perceptual load and valence was not significant (*p* = 0.689).

### 3.2 Time-frequency analysis results

**Figure 4:**
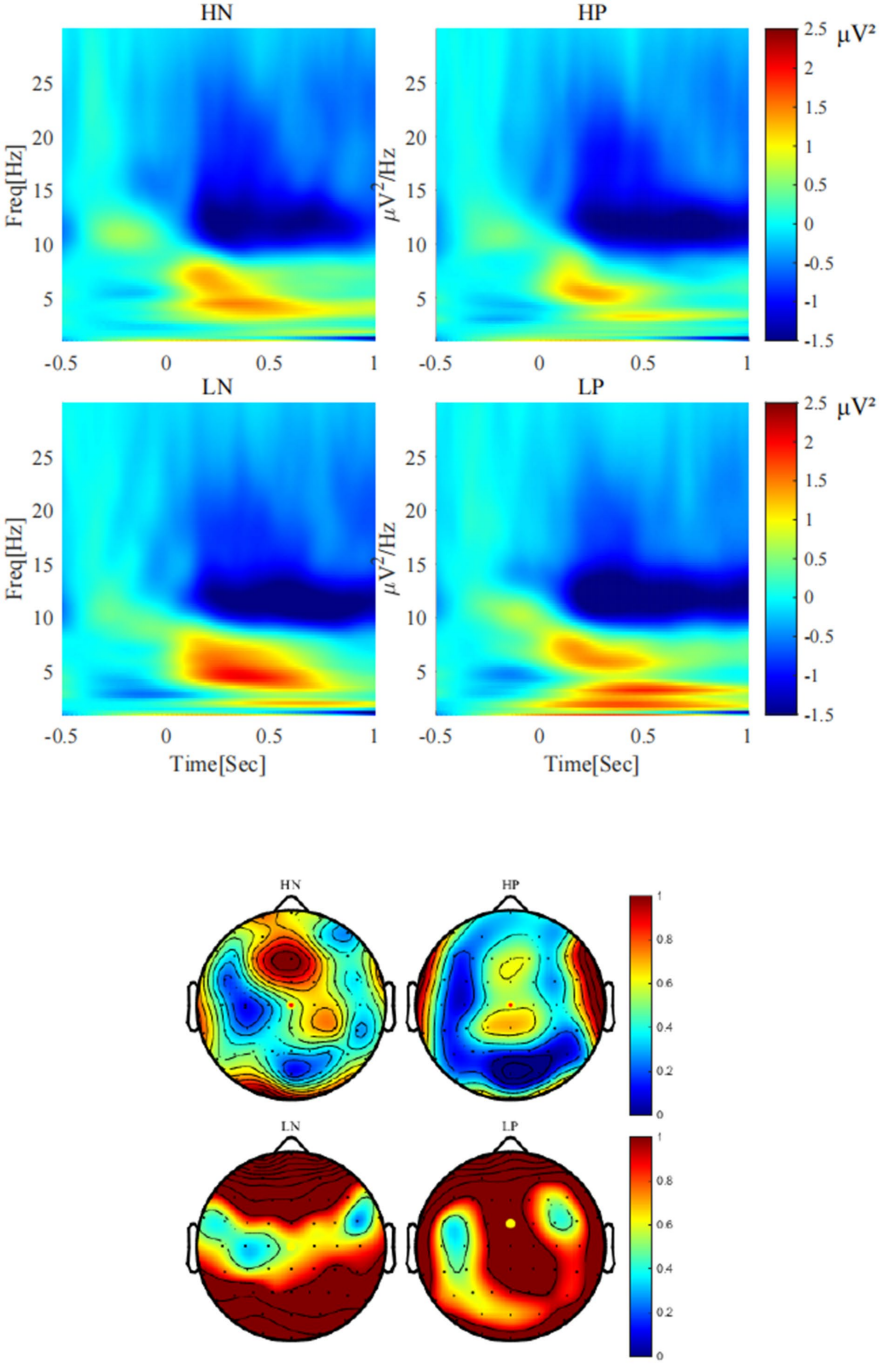
The activities of delta (< 3 Hz) under each condition (Cz).

As shown in Figure 4, for delta, the main effect of valence was significant, *F*(1, 24) = 8.54, *p* = 0.007, *η2 p* = 0.26, with delta activity after positive feedback (0.93 ± 0.16 *μV^2^*) being stronger than that after negative feedback (0.52 ± 0.14 *μV^2^*). The main effect of perceptual load was significant, *F*(1, 24) = 5.55, *p* = 0.027, *η2 p* = 0.19, with delta activity being stronger in the low perceptual load condition (0.97 ± 0.19 *μV^2^*) than in the high perceptual load condition (0.48 ± 0.14 *μV^2^*). The interaction between perceptual load and valence was significant, *F*(1, 24) = 6.32, *p* = 0.019, *η2 p* = 0.21, the simple effect analysis showed that only under the low load condition, there was a significant difference between positive feedback (1.35 ± 0.22 *μV^2^*) and negative feedback (0.58 ± 0.24 *μV^2^*) (*p* = 0.005) conditions.

**Figure 5:**
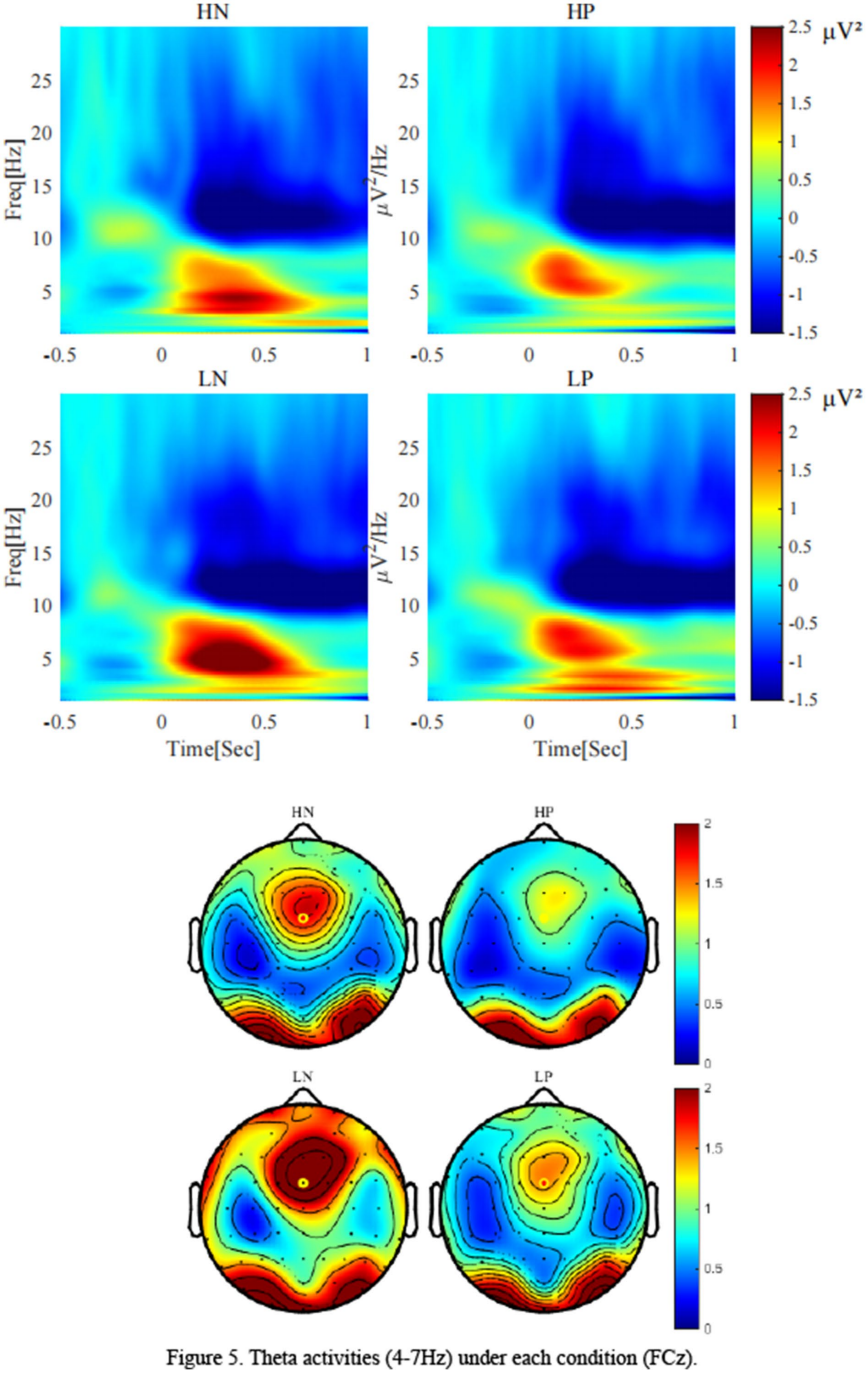
Theta activities (4-7 Hz) under each condition (FCz).

As shown in Figure 5, for theta, the main effect of valence was significant, *F*(1, 24) = 9.79, *p* = 0.005, *η2 p* = 0.29, Theta activity after negative feedback (2.09 ± 0.28 *μV^2^*) was stronger than that after positive feedback (1.29 ± 0.25 *μV^2^*). The main effect of perceptual load was significant, *F*(1, 24) = 12.33, *p* = 0.002, *η2 p* = 0.34, Theta activity was stronger in the low perceptual load condition (2.01 ± 0.27 *μV^2^*) than in the high perceptual load condition (1.37 ± 0.23 *μV^2^*). The interaction between valence and load showed a marginal trend, *F*(1, 24) = 3.32, *p* = 0.081, η2 *p* = 0.12. Simple effect analysis showed that only under the condition of low perceptual load condition, there was significant difference between positive feedback (1.45 ± 0.27 *μV^2^*) and negative feedback (2.57 ± 0.36 *μV^2^*) (*p* = 0.003), while no significant difference was observed under high load.

## 4 Further data analysis

The results of the above ANOVA indicated that perceptual load could modulate only the valence effect of delta, but not those of the RewP, P300 components, or theta oscillation. To explain these results, the psychological functions of these ERP components and oscillations must first be disentangled. To reveal the psychological functions of the four electrophysiological responses, we further analyzed the correlations between RewP and P300, as well as between theta and delta. Additionally, regressions of delta and theta to RewP and P300 were conducted.

### 4.1 Correlation analysis

Since the main effect of valence on both RewP and P300 were significant in the ANOVAs, the difference waves for the valence effect (positive feedback minus negative feedback) were used as indices to analyze the correlations for the valence.

The results are presented in Table 1. For RewP and P300, significant correlations were found for the valence effect. However, no significant correlations were found for delta and theta with respect to valence effect. Delta and RewP were significantly correlated for the valence effect under the low load condition. Theta and P300 were significantly correlated for the valence effect only under the high load condition.

**Table 1:** Correlations of valence effect and load effect between the four components, under four conditions.

	HP - HN	LP - LN
Delta and RewP	0.38^	0.61**
Theta and RewP	0.24	−0.27
Delta and P300	0.32	0.35
Theta and P300	0.41*	−0.08
Delta and theta	0.004	−0.17
RewP and P300	0.93**	0.79**

HP is the high-load positive feedback condition, HN is the high-load negative feedback condition, LP is the low-load positive feedback condition and LN is the low-load negative feedback condition. RewP and delta were marginally significant in the HP - HN condition, *r* = 0.38, *p* = 0.064.

^*p* < 0.1; *p < 0.05; **p < 0.01;***p < 0.001.

Bold values indicate statistical significance.

### 4.2 Regression analysis

The regressions were also only carried out on difference waves, to analyze the contributions of valence effect from oscillations to ERPs.

Table 2 presents the contributions of delta to RewP and theta to P300. The contribution of delta to RewP for the valence effect was significant only under low load conditions. The contribution of theta to P300 for the valence effect was significant only under the high load condition.

**Table 2:** Contribution of the delta and theta to RewP and P300 under four conditions.

		Delta		Theta		Total	
		*Beta*	*t*	*Beta*	*t*	*F*	R^2^
RewP	HP-HN	0.37	1.96^	0.24	1.24	2.71	0.19
	LP-LN	0.57	3.39**	−0.18	−1.06	7.11**	0.39
P300	HP-HN	0.32	1.76	0.41	2.22*	4.04*	0.27
	LP-LN	0.35	1.72	−0.02	−0.12	1.57	0.13

Under HP-HN conditions *t* = 1.96, *p* = 0.063, marginally significant.

^*p* < 0.1; *p < 0.05; **p < 0.01;***p < 0.001.

Bold values indicate statistical significance.

This study specifically investigates the valence effect. The contribution of delta to RewP for the valence effect was significant only under the low load condition, while the contribution of theta to P300 for the valence effect was significant only under the high load condition.

## 5 Discussion

This study manipulated perceptual load to examine its effect on electrophysiological components associated with feedback processing. Both RewP and P300 amplitudes were more positive under low load conditions compared to high load conditions, consistent with previous studies (Krigolson et al., 2012; Liu and Gehring, 2009; Liu et al., 2014; Gehring and Willoughby, 2002; Nieuwenhuis, 2004; Pfabigan et al., 2015; Harmon-Jones et al., 2024). Additionally, the main effect of perceptual load was observed in oscillations, with delta and theta being stronger under low load conditions compared to high load conditions. A straightforward explanation is that more perceptual resources are allocated in low load conditions, which facilitates feedback perception, as evidenced by the enhanced electrophysiological responses. This suggests that all four brain responses are involved in the perception stage of feedback processing, and also indicates that the manipulation of perceptual load is valid in this study.

More importantly, the study found that perceptual load modulated the valence effect of delta, with positive feedback inducing greater delta activity than negative feedback only under low load conditions. For the other components, although the main effect of valence was replicated, no moderating effect of perceptual load on the valence effect was observed. Recent studies have proposed that the RewP reflects reward processing (Holroyd et al., 2008; Foti et al., 2011; Proudfit, 2015), while delta is also considered to reflect positive feedback processing. However, this study found that perceptual load only modulated the valence effect of delta, suggesting that the functions of delta and RewP need to be more precisely distinguished. The studies have analyzed the relationships between oscillations and ERP components (Foti et al., 2015; Bernat et al., 2015; Watts et al., 2017), suggesting that the RewP is a combination of delta and theta activities, reflecting both positive and negative feedback processing, while delta specifically reflects positive feedback processing. This may explain the differential moderating effects of perceptual load on the valence effects of delta and RewP.

To verify the validity of this interpretation, the relationships between oscillations and ERP components were further analyzed. The following first discuss the psychological functions of RewP, P300, delta, and theta, based on the analysis of the relationships between oscillations and ERP components. Subsequently, the effects of perceptual load on these components are explained in light of their psychological functions.

### 5.1 Psychological functions of electrophysiological components related to feedback processing

Initially, researchers considered the RewP to reflect negative feedback processing and characterized it as a negative-going component (Miltner et al., 1997; Gehring and Willoughby, 2002; Nieuwenhuis, 2004; Holroyd and Coles, 2002). However, more recent studies have revised this view, proposing that RewP reflects reward-related neural activity and serves as a marker of reward sensitivity (Holroyd et al., 2008; Foti et al., 2011; Proudfit, 2015; Holroyd and Umemoto, 2016). To reconcile these perspectives, researchers have turned to time-frequency analysis, which reveals that RewP may reflect the summation of distinct oscillatory processes.

Specifically, Bernat et al. (2008, 2011, 2015) demonstrated that delta oscillations are primarily sensitive to positive feedback, while theta oscillations are more responsive to negative feedback. Foti et al. (2015) further localized these oscillations, showing that delta activity originates in the striatum and theta activity in the anterior cingulate cortex (ACC). Based on these findings, it has been proposed that RewP represents a composite signal arising from the integration of delta and theta activity.

In the present study, we replicated these patterns: delta activity increased in response to positive feedback, while theta activity increased following negative feedback. Importantly, the valence effects of delta and theta were not significantly correlated, suggesting that these oscillatory responses reflect functionally distinct processes. Regression analyses further indicated that delta, but not theta, significantly predicted the valence effect of RewP, supporting the interpretation that RewP in this study predominantly reflects positive feedback processing.

Turning to the P300, it has traditionally been associated with the allocation of attentional resources, context updating, and working memory processes (Polich, 2007). In feedback learning paradigms, the P300 has also been linked to the motivational significance of outcomes (Nieuwenhuis et al., 2005). While many studies, including ours, have reported a valence effect on P300 (i.e., greater amplitude for positive compared to negative feedback; Zhou et al., 2010; Leng and Zhou, 2010), this does not necessarily imply that P300 exclusively reflects positive feedback processing.

Notably, our regression analysis revealed that theta activity—typically associated with negative feedback, conflict monitoring, and cognitive control—significantly predicted P300 amplitude under high perceptual load. This suggests that, particularly in cognitively demanding situations, P300 may integrate signals from loss-related processing to support adaptive attention or behavioral adjustment.

Therefore, we interpret the P300 not as a unidimensional marker of feedback valence, but rather as a dynamic integration signal, sensitive to the task context, cognitive load, and motivational relevance of outcomes. This interpretation aligns with the valence-agnostic perspective proposed by Polich (2007) and provides a more comprehensive framework for understanding the flexible role of P300 in feedback processing.

### 5.2 Effects of perceptual load on feedback processing

The primary goal of feedback processing is to determine whether behavior is correct and to adopt appropriate strategies for maintaining or adjusting behavior. Accordingly, this study focused on the valence effect, with particular emphasis on the moderating role of perceptual load in modulating feedback-related neural responses.

Our findings indicate that perceptual load modulates multiple stages of feedback processing, through distinct mechanisms. Specifically, perceptual load selectively influenced the valence effect of delta oscillations, which are sensitive to positive feedback processing. Under low-load conditions, delta activity increased in response to positive feedback, facilitating its discrimination and encoding. However, under high-load conditions, delta activity was suppressed, likely reflecting a diminished ability to process positive feedback, which may in turn impair learning from rewarding outcomes.

Although RewP is also known to reflect reward sensitivity, perceptual load did not directly modulate the valence effect of RewP in our data. One possible explanation is that RewP reflects a mixture of neural signals associated with both positive reward and omission of expected reward (Foti et al., 2015; Bauer et al., 2024), whereas perceptual load may influence only the purely positive feedback component (i.e., delta), not the omission-related component (i.e., theta). As a result, the modulation effect of perceptual load on RewP may be diluted or masked at the averaged ERP level. This interpretation is consistent with prior studies showing that the valence effect of RewP decreases under perceptually challenging conditions (Cockburn and Holroyd, 2018; Gibbons et al., 2016; Krigolson et al., 2012; Liu and Gehring, 2009; Mars et al., 2004; Pfabigan et al., 2014, 2015). These studies, along with our findings, suggest that RewP amplitude attenuation under high load may be primarily driven by a reduction in delta activity.

Further evidence supporting this interpretation comes from our regression analyses. We found that the valence effect of delta significantly predicted RewP, but only under low-load conditions, suggesting that delta-driven RewP effects are contingent on available perceptual resources. In contrast, theta activity, typically linked to negative feedback and conflict monitoring, significantly predicted P300 amplitude under high-load conditions, but not under low load. This suggests that P300 integrates loss-related signals to support behavioral regulation, particularly when perceptual demands are high.

Moreover, although both delta and theta contributed to ERP components associated with feedback processing, perceptual load only moderated the delta-to-RewP relationship, but not the delta-to-P300 or theta-to-P300 associations. This dissociation suggests that RewP is more directly tied to the perceptual stage of processing, which is susceptible to load-induced constraints. By contrast, P300 appears to reflect a later stage of feedback evaluation, involving cognitive control and adaptive response selection, which may be more resilient to perceptual interference.

Together, these findings highlight the differential roles of delta and theta oscillations in shaping feedback-related ERP components and underscore the stage-specific influence of perceptual load: it disrupts early valence-specific encoding (RewP) by taxing perceptual resources, but has less impact on later cognitive integration processes (P300), which engage compensatory mechanisms such as attentional control.

### 5.3 Study limitations

Although the present study employed a simple gambling task in which feedback was randomized and carried no actionable value, our findings offer meaningful insights into the early neural mechanisms of feedback processing that are relevant for learning. Due to the lack of learning contingencies, higher-order behavioral adjustments were not required, and the task did not engage goal-directed adaptation. Nevertheless, the modulation of RewP and delta activity by perceptual load suggests that these components may serve as early indicators of reward salience and perceptual accessibility—both of which are foundational for effective learning.

Beyond the framework of reward sensitivity and the PRO (prediction of response-outcome models) theory, these findings align with principles from reinforcement learning (RL) models, which propose that prediction error signals—reflecting the discrepancy between expected and actual outcomes—drive learning. The RewP has been widely interpreted as an ERP correlate of reward prediction error (Holroyd and Coles, 2002), and our results suggest that delta oscillations may represent an early-stage, bottom-up component of this signal, particularly when perceptual demands are low and feedback is more easily processed.

Moreover, in the context of perceptual resource theory (Lavie, 2006), the enhanced delta and RewP responses under low-load conditions support the view that attentional capacity influences the efficiency of feedback processing. When perceptual complexity is reduced, more cognitive resources are available to encode reward-related signals, leading to more robust neural differentiation between feedback valence conditions. This underscores the role of perceptual gating as a modulator of early reward evaluation processes.

In addition, theta oscillations, although not strongly modulated by load in this study, may reflect a positive feedback omission–adjustment process, consistent with the PRO theory (Alexander and Brown, 2010, 2011), which emphasizes the role of medial frontal theta in signaling expectation violations that can guide future behavior.

To further evaluate these interpretations and build a more comprehensive account of feedback-guided learning, future studies should employ paradigms that integrate true learning demands, such as reinforcement learning or probabilistic choice tasks, and systematically vary perceptual load. Such designs would clarify how perceptual complexity shapes the neural dynamics of feedback processing and behavioral adaptation over time, and reveal how early-stage ERP and oscillatory markers contribute to learning-related decision updating.

## Data Availability

The raw data supporting the conclusions of this article will be made available by the authors, without undue reservation.
